# Dual Specificity Phosphatase 5, a Specific Negative Regulator of ERK Signaling, Is Induced by Serum Response Factor and Elk-1 Transcription Factor

**DOI:** 10.1371/journal.pone.0145484

**Published:** 2015-12-21

**Authors:** Camille Buffet, Maria-Grazia Catelli, Karine Hecale-Perlemoine, Léopoldine Bricaire, Camille Garcia, Anne Gallet-Dierick, Stéphanie Rodriguez, Françoise Cormier, Lionel Groussin

**Affiliations:** 1 Endocrinology-Metabolism-Diabetes Department, Institut Cochin, Université Paris Descartes, CNRS (UMR8104), INSERM U1016, Paris, France; 2 Department of Endocrinology, Cochin Hospital, Paris, France; University of Louisville, UNITED STATES

## Abstract

Serum stimulation of mammalian cells induces, *via* the MAPK pathway, the nuclear protein DUSP5 (dual-specificity phosphatase 5), which specifically interacts with and inactivates the ERK1/2 MAP kinases. However, molecular mechanisms underlying *DUSP5* induction are not well known. Here, we found that the *DUSP5* mRNA induction depends on a transcriptional regulation by the MAPK pathway, without any modification of the mRNA stability. Two contiguous CArG boxes that bind serum response factor (SRF) were found in a 1 Kb promoter region, as well as several E twenty-six transcription factor family binding sites (EBS). These sites potentially bind Elk-1, a transcription factor activated by ERK1/2. Using wild type or mutated *DUSP5* promoter reporters, we demonstrated that SRF plays a crucial role in serum induction of *DUSP5* promoter activity, the proximal CArG box being important for SRF binding *in vitro* and in living cells. Moreover, *in vitro* and *in vivo* binding data of Elk-1 to the same promoter region further demonstrate a role for Elk-1 in the transcriptional regulation of DUSP5. SRF and Elk-1 form a ternary complex (Elk-1-SRF-DNA) on *DUSP5* promoter, consequently providing a link to an important negative feedback tightly regulating phosphorylated ERK levels.

## Introduction

Mitogen-activated protein kinases (MAPK) cascades are a conserved group of signal transduction pathways responsible for the transduction of various signals to a large number of cellular protein substrates [1]. The MAPK pathway, culminating in the activation of extracellular signal-related kinases (ERK1/2) by the MAPK kinase MEK, consists in a cascade of phosphorylation lying downstream of the cellular proto-oncogene RAS thus eliciting cellular responses like proliferation, differentiation, transformation, and survival. ERK1 and ERK2 isoforms are both phosphorylated at the conserved T-X-Y motif in the activation loop of the kinase. ERK is subject to negative regulation by specific protein phosphatases. Among them, two dual-specificity (Thr/Tyr) MAPK phosphatases (DUSPs), DUSP5 and DUSP6, localized in the nucleus [2] and cytoplasm, respectively [3], specifically dephosphorylate ERK [2, 4, 5]. These phosphatases belong to the large family of DUSPs, so-called as they dephosphorylate both tyrosine and serine/threonine residues [6]. The binding of DUSP6 to ERK is associated with catalytic activation of the bound phosphatase and can play a role in cytoplasmic retention of inactivated ERK through its NES (nuclear export signal) [7–9]. On the contrary, DUSP5 activity seems unaffected upon ERK binding and phosphorylation and its basal activity in the absence of ERK activation is greater than that of DUSP6 [2]. Thus, since DUSP5 possesses a functional NLS (nuclear localization signal) and has been proposed to act as a nuclear anchor for ERK, its substrate selectivity is only determined by the specific interaction with nuclear ERK [2].

DUSP5 and DUSP6 are known to be induced by ERK signaling [10–12], and thereby are involved in a negative feedback loop that tightly controls phosphorylated ERK (pERK) levels. The role of DUSPs in both cancer progression and cancer resistance becomes obvious, making them rational targets for new therapeutics [13]. In differentiated thyroid cancer, a tumorigenesis model studied in our laboratory, the MAPK pathway is constitutively activated [14]. Some DUSPs have been shown to be significantly up-regulated, compared to normal thyroid tissue [15] and are supposed to be a marker of high-risk feature in such tumors [16]. A recently published transcriptome analysis of 496 papillary thyroid cancers confirmed that cancers with the most robust activation of MAPK signaling presented high levels of DUSP4, DUSP5 and DUSP6 mRNAs [17]. Modulation of *DUSP5* expression has been shown to alter the decision of growth arrest versus proliferation of human cancer cells [18, 19].

Mechanisms regulating *DUSP6* expression have been largely elucidated, contrary to those controlling *DUSP5* expression. It has been shown that *DUSP6* is regulated by the MAPK pathway, at the transcriptional level, through a conserved binding site for transcription factors of the E twenty-six family (ETS) Ets-1 and Ets-2, within a 508 bp promoter region. [11, 12, 20]. Ets-1 and Ets-2 are well known direct targets of the MAPK pathway, as most of the ETS transcription factors [21]. The highly conserved ETS binding site (EBS) containing an invariable core motif, 5’-GGA(A/T)-3’, defines this family of transcription factors, including Ets-1, Ets-2 and Elk-1. For the *DUSP6* gene, Ets-1 and Ets-2 are supposed to be bound to their responsive element in the basal state, presumably associated with co-repressors [22]. ERK-phosphorylation of specific residues in the N terminal region of Ets-1 and Ets-2 could lead to the binding of a co-activator (such as CBP/p300) and to an increased transcription of target genes [22–24]. *DUSP6* not only is regulated by the MAPK pathway at the transcriptional level but also at the post-transcriptional level as MEK-ERK pathway has been shown to stabilize *DUSP6* mRNA [25].

Concerning *DUSP5*, one previous study has demonstrated in a human colon-cancer cell line that p53 could bind to a sequence located approximately 1.2 kb upstream of the transcription start site and induce *DUSP5* expression [19]. Nevertheless regulation of *DUSP5* by p53 does not explain how MAPK pathway activation is responsible for the induction of *DUSP5* expression.

The purpose of this paper was to determine the precise mechanism of regulation of the *DUSP5* gene by the MAPK pathway at the transcriptional and/or post-transcriptional level. Bioinformatic analysis allowed us to identify many EBS, putatively binding Elk-1, a member of the ternary complex factors (TCF) sub-family of ETS transcription factors, as well as binding sites for the serum response factor (SRF), namely CArG boxes, in a ~ 1kb promoter region of *DUSP5*. The combination of one EBS and one CArG box corresponds to a serum responsive element (SRE). In the present work we found that *DUSP5* mRNA is a short-lived messenger rapidly induced by ERK activation and that its mRNA stability is independent from the activation of the MAPK pathway, unlike DUSP6. Different experimental approaches were used to understand the role of the regulatory components of the *DUSP5* promoter. Our findings indicate altogether that the ternary complex SRF-Elk-1-SRE is crucial in regulating *DUSP5* transcription, providing a mechanistic link between MAPK pathway signaling and *DUSP5* induction.

## Experimental Procedures

### Reagents

Actinomycin D and cycloheximide were purchased from Sigma-Aldrich. Wortmannin was from Calbiochem. The MEK inhibitor UO126 was from Promega. Disuccinimidyl glutarate (DSG) was from SantaCruz Biotechnology.

### Cell lines

NIH/3T3 (mouse fibroblast) cell line was purchased from ATCC (American Type Culture Collection) and maintained in Dulbecco’s Modified Eagle’s Medium, containing 10% foetal calf serum (FCS) and antibiotics. Serum starvation and stimulation consisted of maintaining cells in respectively 0.25% or 20% FCS.

### Quantitative reverse transcription-PCR assay

Total RNA was extracted from cultured cells using the RNeasy Mini Kit from Qiagen and was reverse transcribed to generate cDNA (High-Capacity^®^ cDNA Reverse Transcription Kit, Applied Biosytem cat # 4368814). Real-time PCR was done using a light cycler instrument (LightCycler^®^ FastStart DNA Master SYBR Green I, *Roche*). The relative gene expression was calculated using the 2^-ΔC^_T_ method.

### Immunoblotting

Whole cell lysates were analyzed by Western Blotting. Briefly, cells were lysed in 50mM TrisHCl (pH 7.5)/ 1mM EDTA/ 150mM NaCl/ 1% Nonidet P-40 and a cocktail of protease and phosphatase inhibitors (Complete protease inhibitor cocktail tablets and PhosSTOP phosphatase inhibitor cocktail tablets; Roche Diagnostics). Protein concentration was determined using the Bradford reagent (Bio-Rad). Twenty μg of lysate were subjected to SDS–PAGE on 10% acrylamide gels and transferred onto nitrocellulose membranes (Amersham Pharmacia Biotech). Nonspecific protein-binding sites were blocked by incubation for 1 hour at room temperature in 50mM Tris-HCl (pH8), 150mM NaCl and 0.1% Tween 20 (TBS-T) containing 10% nonfat dry milk. Incubation with primary antibodies was carried in the same buffer overnight at 4°C. Antibodies directed against pERK from Santa Cruz Biotechnology (1:1000), Inc. (sc-7383); total ERK (1:10000) from Cell Signaling Technology^®^ (#9102), pAKT (1:1000) from Cell Signaling Technology^®^ (#9271), t-AKT (1:1000) from Cell Signaling Technology^®^ (#9272), and GAPDH (1:2000) from Santa-Cruz Biotechnology (sc-25778) were used. Secondary antibodies used were peroxidase-conjugated antirabbit IgG at 1:10000 or peroxidase-conjugated antimouse at 1/10000. The specific complexes were detected using the enhanced chemiluminescence (ECL) system from Amersham Pharmacia Biotech.

### Plasmid constructs for cellular transfection

A promoter region of 975 base-pair of the *DUSP5* gene, containing a putative TATA box, was isolated by PCR from a rat bacterial artificial chromosome (BAC) CH230-312K10 (CHORI.ORG) and sub-cloned into pGL3b plasmid (Promega) containing the reporter firefly luciferase. Three shorter reporter plasmids bearing different lengths of *DUSP5* promoter region were then produced. Responsive elements in the shortest plasmid (165 bp) were mutated at various sites with the QuikChange^®^ Site-Directed Mutagenesis Kit (Stratagene).

Plasmids SRF-VP16, Elk-VP16, Elk-En, and Elk-VP16 (L158P) were a gift from A.D. Scharrocks and E.R. Vickers [26, 27]. SRF-En was a gift from B. Knöll [28]. Elk-1-HA was a gift from P. Vanhoutte [29]. Plasmid expressing SRF was a gift from A. Sotiropoulos [30]. Cellular transient transfection was performed with Lipofectamine Plus (Invitrogen). Luciferase assays were performed in at least triplicate. A representative experiment of at least three independent ones is presented.

### Small interference RNA transfection

Small interfering RNA (siRNA) were purchased from Eurofins MWG Operon. Two mouse *SRF* or two *Elk-1* siRNA were transfected simultaneously at the concentration of 50 nmol/L each with Lipofectamine Plus (Invitrogen) in three independent experiments. A scramble siRNA was used as control (5'-GCCACTACCTCGTTTCACA-3'). After transfection, the level of mRNA knockdown was assessed by reverse transcription quantitative PCR.

### Electrophoretic mobility shift assay (EMSA)

EMSA was performed using nuclear extracts of NIH/3T3 cells, different ^32^P-labeled fragments of the *DUSP5* promoter and of an unrelated probe. To demonstrate the specificity of DNA/protein complex formation, a 20-fold molar excess of the various unlabeled probes was used. To demonstrate the presence of a specific protein in the complexes, nuclear extracts were mixed with 2 μg of the following antibodies: SRF from Santa Cruz Biotechnology, Inc. (sc-13029X), HA-probe from Santa Cruz Biotechnology, Inc. (sc-805X), or rabbit Ig G from Santa Cruz Biotechnology, Inc. (sc-2027). A representative experiment of at least three independent ones is presented.

### Chromatine Immunoprecipitation

NIH/3T3 cells were serum starved for 24 hours and then stimulated with 20% FCS for 30 minutes. A two-step cross linking procedure was used: incubation with 2 mM of disuccinimidyl glutarate (DSG) for 45 minutes prior to cross-linking with formaldehyde (1%) for 10 minutes at room temperature. Cross-linking was stopped by adding glycine and the cells lysed. Nuclear lysates were sonicated under conditions yielding fragments ranging from 200 to 800 bp. ChIP was performed by using the ChIP-Adem-Kit (Ademtech) and the automated purification system “KingFisherDuo” (ThermoFisher). Cross-linking was reversed by incubation at 65°C (5 hours or overnight). Antibodies against Elk1 and SRF from Santa Cruz Biotechnology and an antibody against Elk1 from Millipore were used. After digestion with Proteinase K (10mg/ml, 2 hours at 37°C), DNA was purified and used for qPCR. The results were reproduced in three independent experiments. The following mouse oligonucleotides were used for qRT-PCR in ChIP assays: DUSP5 intron 3: 5’-GAGACTGAGGGTGGCAAGAG (forward primer) and 5’-ACTGGCTGTGAGCACGTATG (reverse primer) and *DUSP5* promoter: 5’-CCACTTCCTCTTTCTCGCTCT (forward primer) and 5’CGCAGGGTTTTATGTGAATG (reverse primer).

### Statistical analysis

Statistical significance was determined using the Student’s test (online GraphPad Software).

## Results

### Induction and half-life of *DUSP5* mRNA

**Fig 1:**
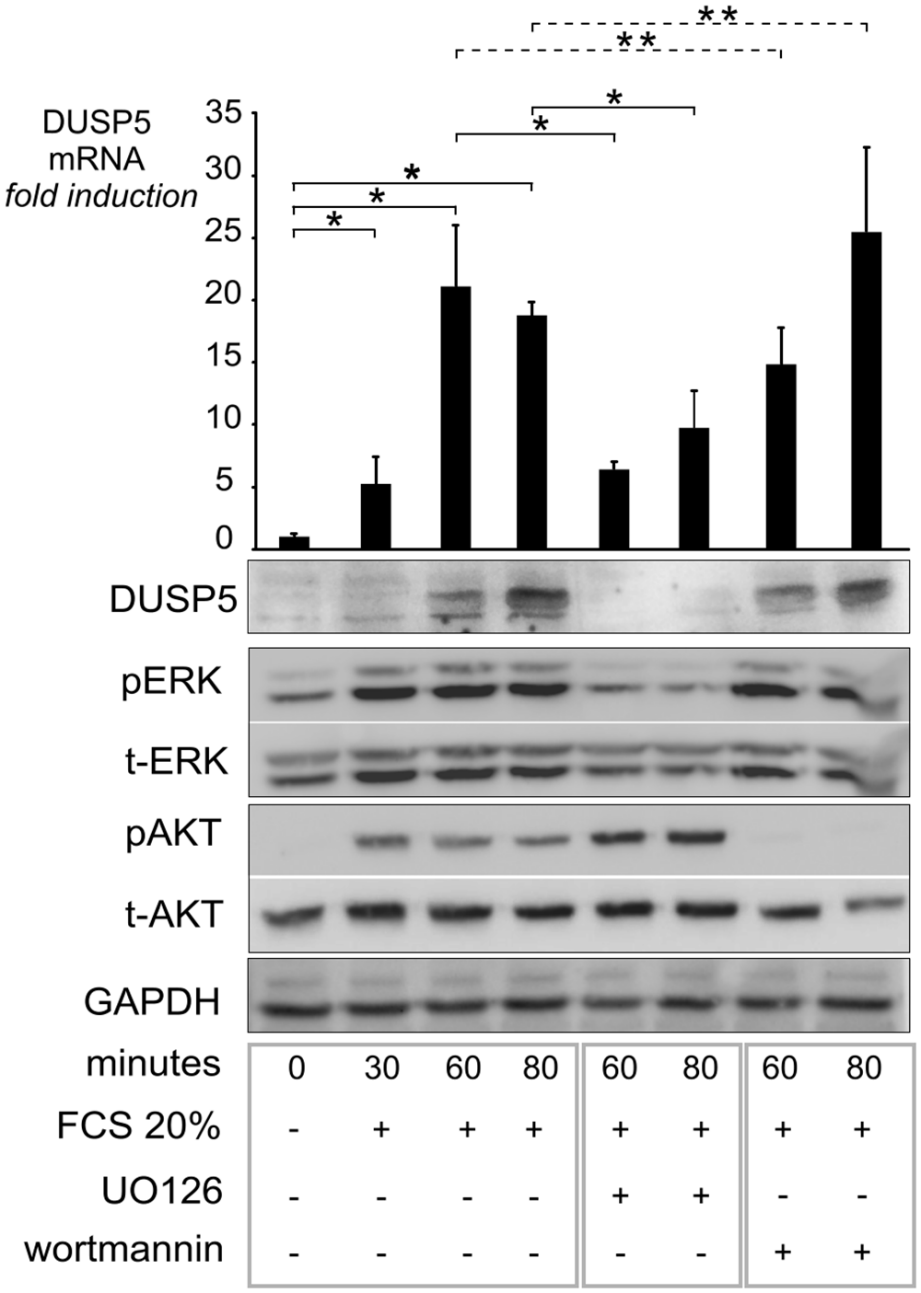
*DUSP5* is an early response gene induced by ERK signalling. NIH/3T3 cells were stimulated by FCS 20% and treated either with 20 μM UO126 (MEK inhibitor) or 80nM wortmannin (PI3K inhibitor) for the indicated time. *DUSP5* mRNA levels were measured by RT-qPCR and normalized for cyclophilin mRNA levels. *DUSP5* mRNA levels at baseline were set at 1 and values at subsequent time points are indicated as fold induction compared to baseline. Protein expression levels were assayed by immunoblot for phosphorylated ERK (p-ERK), total ERK (t-ERK), phosphorylated (p-AKT) and total AKT (t-AKT). The effect of inhibitors on p-ERK and p-AKT levels is shown. * *P* < 0.05; ** *P* = non-significant.

To study the induction of *DUSP5* mRNA, we first evaluated the response of *DUSP5* gene to extracellular signals (Fig 1). NIH/3T3 cells were serum deprived for 12 hours and then stimulated by 20% FCS up to 80 minutes. A rapid increase of *DUSP5* mRNA level was observed after 30 minutes (five-fold, *P* = 0.02), with a further increase of about twenty fold at 60 (*P* = 4.10^−3^) and 80 minutes (*P* < 1.10^−4^), in parallel with an increased ERK phosphorylation. The *DUSP5* mRNA increase was inhibited by 50 to 70% in the presence of UO126 (MEK inhibitor) and not significantly affected (about 30% decreased at 60 min and 30% increased at 80 min) by the PI3K inhibitor wortmannin. We confirmed at the protein level, that DUSP5 increased in parallel with the serum stimulation and decreased with MEK-15344021ERK inhibition. On the opposite, DUSP5 protein levels were not affected by the PI3K inhibitor. We can conclude that *DUSP5* is an early response gene and that its induction by FCS in NIH/3T3 cells is essentially dependent on the MEK/ERK pathway.

**Fig 2:**
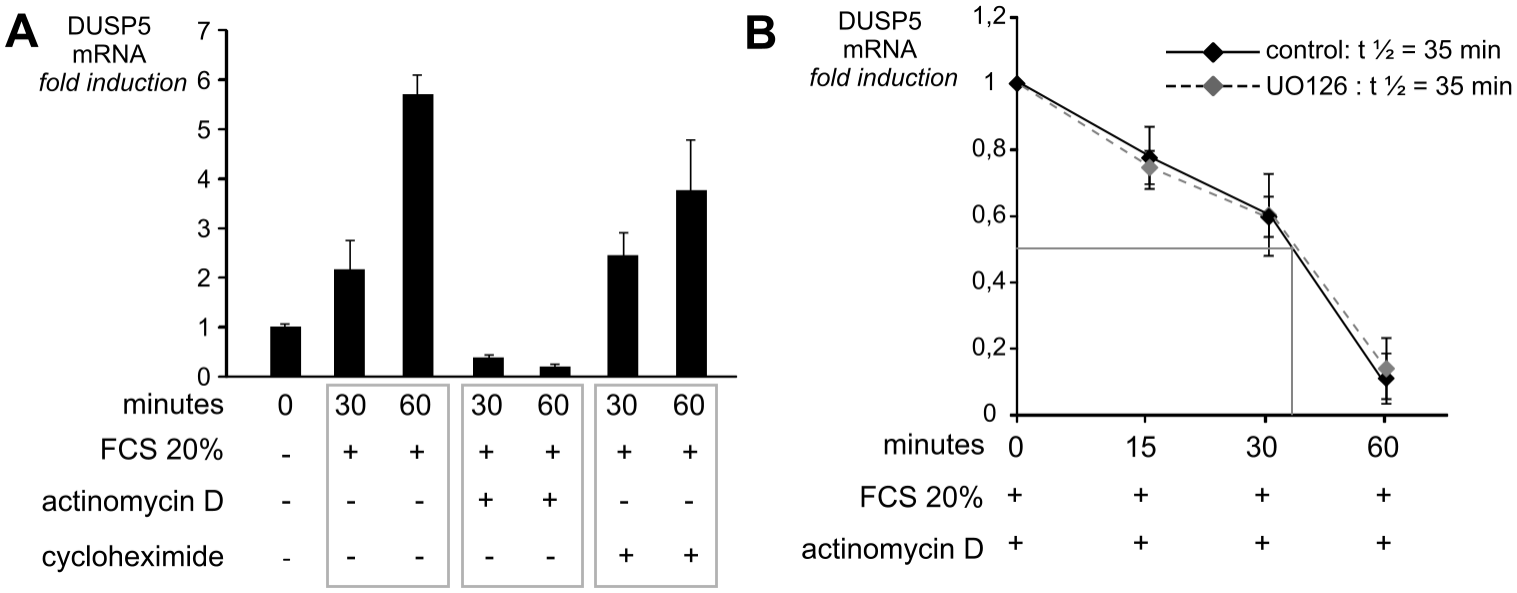
*DUSP5* expression is regulated at the transcriptional level. (A) NIH/3T3 cells were treated with FCS 20% alone or in combination with the transcriptional inhibitor actinomycin D (5 μg/ml) or the protein synthesis inhibitor cycloheximide (100 μg/ml) for the indicated times. *DUSP5* mRNA levels were measured by RT-qPCR and normalized for cyclophilin mRNA levels. *DUSP5* mRNA levels at baseline were set at 1 and values at subsequent time points are indicated as fold induction compared to baseline. * *P* < 0.05, ** *P* = non-significant. (B) NIH/3T3 cells were stimulated with 20% FCS for one hour and then were treated with 5 μg/ml of actinomycin D with or without 20μM of UO126 (MEK inhibitor) for the indicated times. *DUSP5* mRNA levels, before actinomycin D treatment, were taken as 100%. *DUSP5* mRNA levels were measured at different times after UO126 treatment. *DUSP5* mRNA half-life (t ½) is indicated in cells with and without (control) UO126 treatment.

To test whether the rapid and consistent accumulation of *DUSP5* mRNA after serum stimulation was essentially due to an activation of transcription or was dependent on the mRNA stability, the serum treatment of NIH/3T3 cells was combined with low dose actinomycin D (transcriptional inhibitor) or cycloheximide (protein synthesis inhibitor) treatment. While actinomycin D efficiently inhibited the increase of *DUSP5* mRNA level, cycloheximide treatment had no effect (Fig 2A). These data are in favor of a regulation mainly at the transcriptional level. To evaluate if *DUSP5* mRNA was stabilized by the MAPK pathway activation, NIH/3T3 cells were stimulated with 20% FCS for one hour, then the half-life of *DUSP5* mRNA was calculated in the presence of actinomycin D with or without UO126. Results presented in Fig 2B indicate that *DUSP5* mRNA is a short-lived messenger (t ½ = 35 min) which is not stabilized by ERK activation, unlike *DUSP6* [25].

### Regulatory regions of *DUSP5* promoter

**Fig 3:**
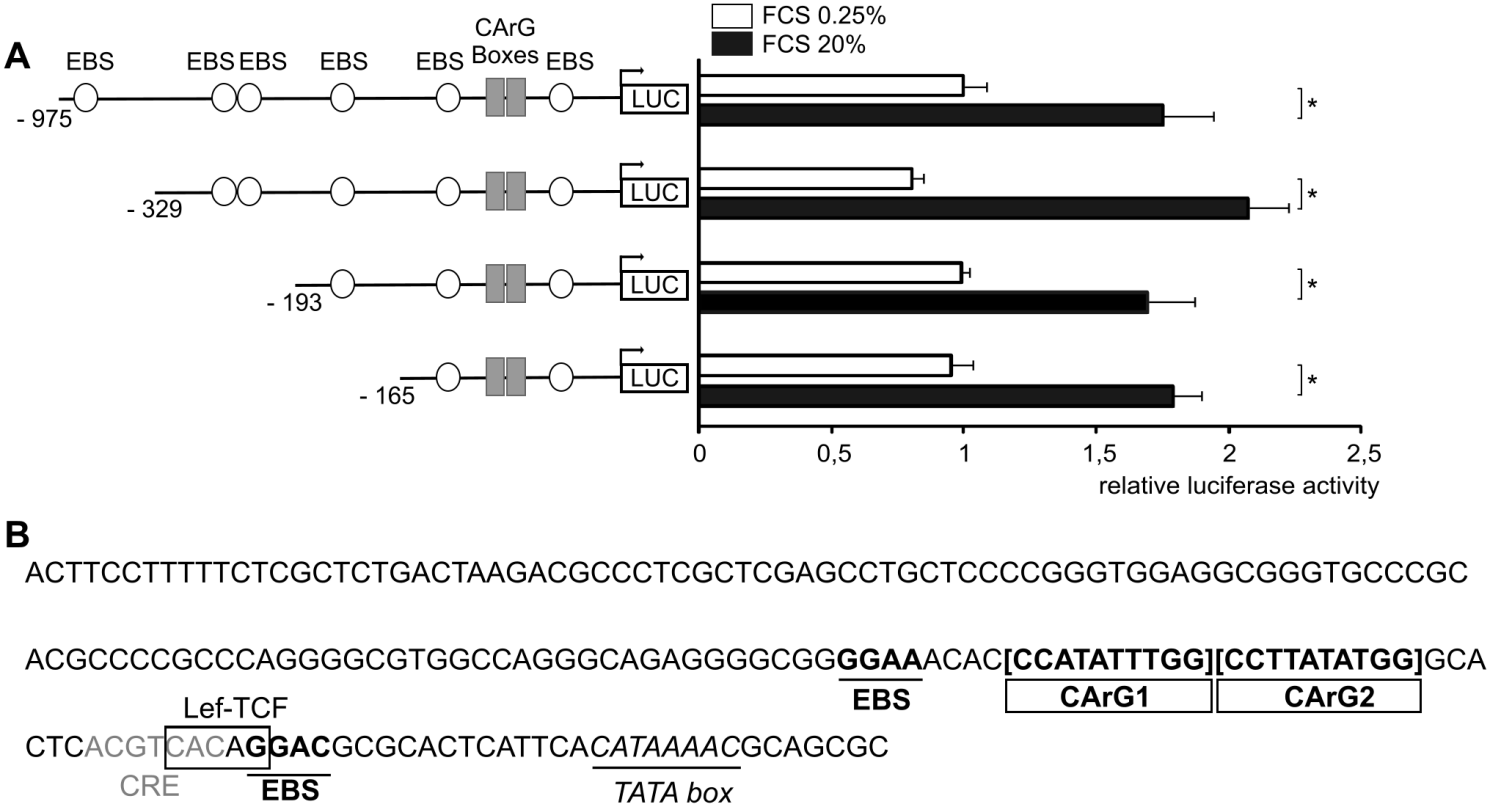
The proximal region of *DUSP5* promoter is sufficient for its serum induction. (A) Reporter vectors harboring full-length or various truncations of the promoter region of *DUSP5* (250 ng) were transfected in NIH/3T3 cells with (black boxes) or without (white boxes) stimulation by FCS 20% for nine hours (Luciferase activity was normalized to Renilla activity). Basal luciferase activities were related to that of full-length construct. Induced luciferase activities of each vector were reported to their own basal activity. * *P* < 0.05. (B) The sequence of the putative proximal promoter region of *DUSP5* gene is shown. Putative transcription factor binding sites are indicated: two contiguous CArG boxes potentially implicated in binding of SRF, two EBS sequences GGA(A/C) potentially implicated in binding of Elk-1, one putative Lef-TCF binding site (Wnt/ β-catenin pathway), and one cAMP response element (CRE).

To understand the mechanisms of transcriptional regulation of the *DUSP5* gene, a search for transcription factors binding sites in the promoter sequence using the Transcription Element Search System (TESS) program from the Department of Biology of the University of Pennsylvania (http://www.cbil.upenn.edu/cgi-bin/tess/tess) revealed the presence of many core sequences GGA(A/T) or GGA(A/C) potentially implicated in the binding of ETS-domain transcription factors (EBS) [21]. We hypothesized that binding sites for ETS-domain transcription factors, well known targets of the MAPK pathway [21], were involved in the transcriptional regulation of *DUSP5* gene. To test this hypothesis and in order to determine the core regulatory region of the promoter, EBS located in the 5’ region of the sequence were progressively deleted to obtain the four constructs represented in Fig 3A. Basal luciferase activities of these four reporter constructs in serum-deprived NIH/3T3 cells were similar. Serum-induced luciferase activities were significantly increased compared to basal values (*P* <0.05). An identical level of stimulation was observed with the four constructs. Within the limits of transfection experiments, this suggests that the shortest *DUSP5* promoter construct is sufficient to elicit a significant serum response, excluding a major role for sequences upstream the nucleotide -165 in transcriptional regulation. These induction levels (i.e. between 2 and 4) have already been observed for the *DUSP6* promoter [12, 20]. ERK drives the upregulation of DUSP5 and DUSP6, which in turn dephosphorylates and thus inactivates ERK [2, 31]. When ERK signaling is turned off, *DUSP5* and *DUSP6* expression decreases. These low induction levels of *DUSP5* and *DUSP6* can be explained by this negative-feedback mechanism. Similar experiments were conducted in the rat pheochromocytoma-derived PC12 cell line, with nerve growth factor (NGF) as stimulation. Identical results were observed (data not shown).

Altogether, these results suggest that the proximal part of the *DUSP5* promoter is sufficient to enable activation of transcription by serum and growth factors, such as NGF, known to activate the MAPK pathway.

The sequence of the proximal promoter region is reported in Fig 3B and putative regulatory sites are indicated. Further analysis of this region revealed the presence of sequences of interest for the regulation of early response gene: two contiguous putative binding sites for SRF, namely CArG boxes. A putative TATA box, corresponding to an alternative of classical TATA box [32], located -22 base pairs upstream from the transcription start site is underlined.

### Role of SRF and Elk-1 transcription factors

**Fig 4:**
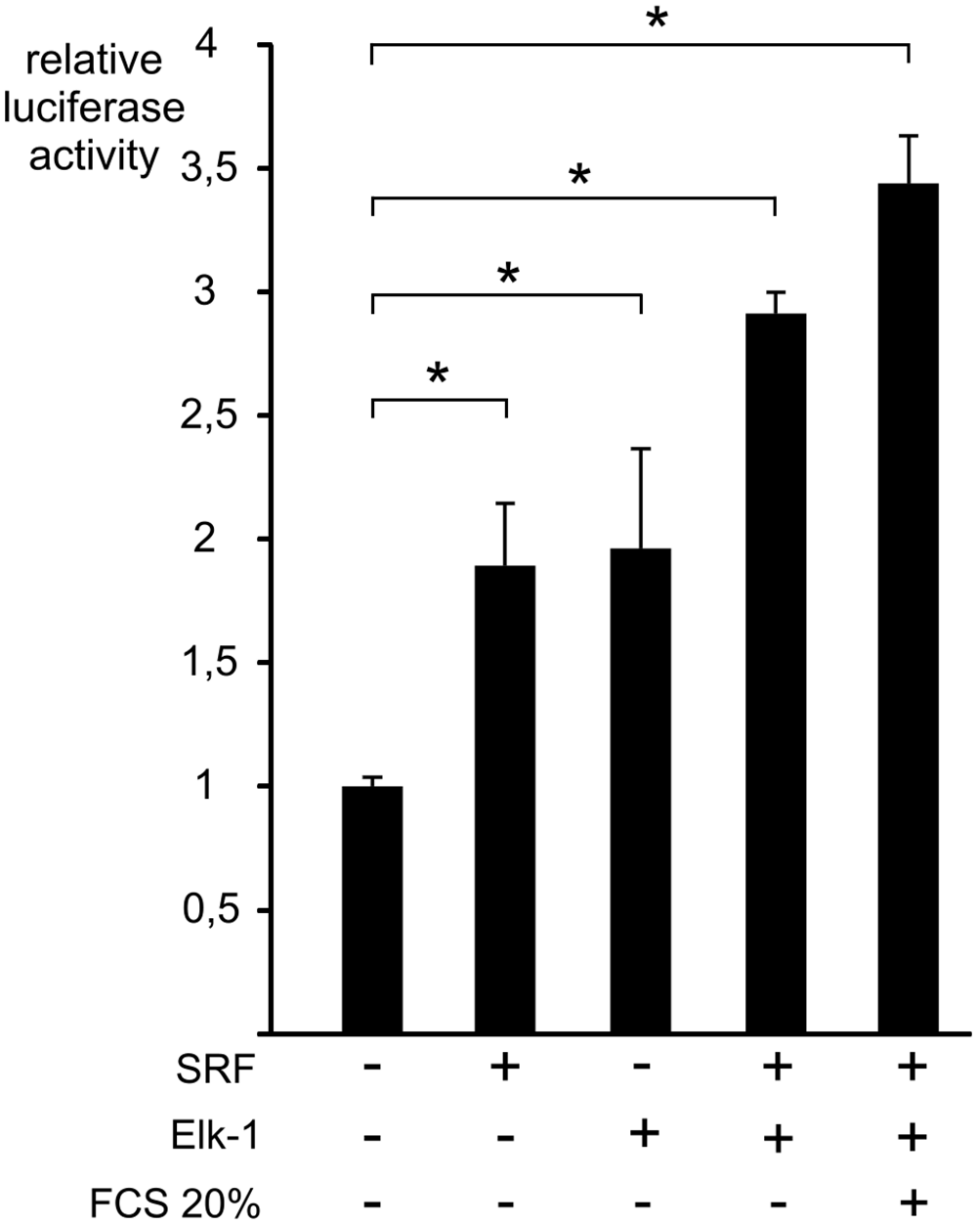
Induction of *DUSP5* promoter by transcription factors SRF and Elk-1. NIH/3T3 cells were transiently transfected with 250 ng of *DUSP5* proximal promoter reporter alone or in combination with SRF (100 ng) or Elk1-HA (300 ng) expression vectors. Luciferase assays were performed in sixplicate and mean values ± S.D. are shown. Cells were starved (0.25% FCS) for 24 hours and then stimulated or not with 20% FCS for nine hours before assessment of luciferase activity. Luciferase activities were reported to the basal luciferase activity of DUSP5 reporter vector without SRF, Elk-1 vectors and FCS stimulation. * *P* < 1.10^−3^.

**Fig 5:**
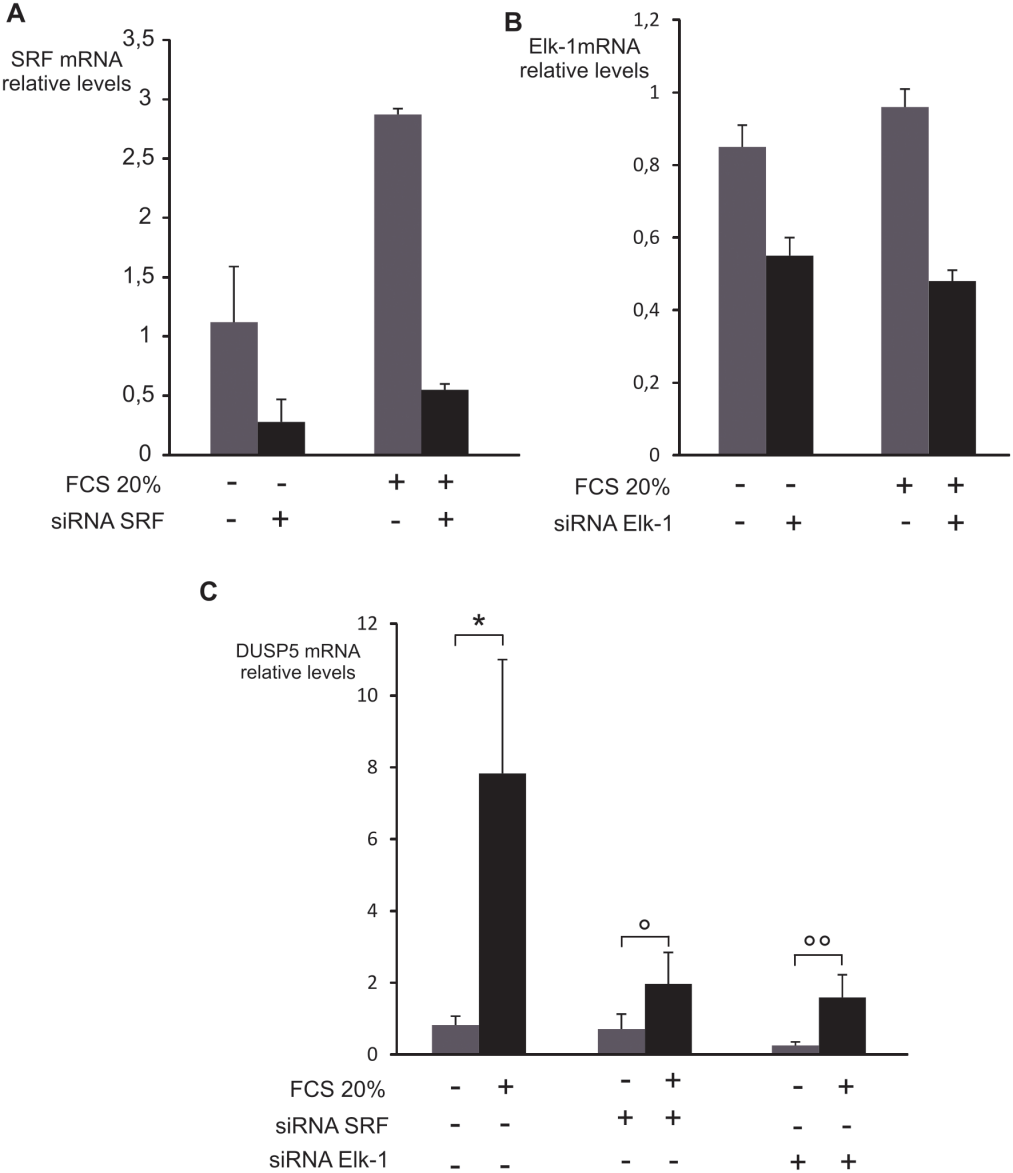
Depletion of endogenous SRF or Elk-1 decreases DUSP5 mRNA induction by serum stimulation. NIH/3T3 cells were transfected with small interfering RNA directed against SRF or Elk-1 or control siRNA. Cells were serum deprived for 12 hours and then stimulated or not with 20% FCS for one hour. To quantify the gene-silencing efficiency by siRNA, SRF (A) and Elk-1 (B) mRNA levels were measured by RT-qPCR and normalized for cyclophilin mRNA levels. (C) DUSP5 mRNA relative levels were compared between the situations with control siRNA and SRF or Elk-1 specific siRNA. The results represent the mean of at least two independent experiments each performed in triplicate ± standard error. * *P* = 0.04; ° *P* = 0.8 °° *P* = 0.05.

Among transcription factors binding to EBS, Elk-1 binding sites are known to co-localize frequently with CArG box [33]. To test the implication of SRF and Elk-1 in *DUSP5* promoter regulation, the shortest *DUSP5* promoter reporter was transfected with plasmids expressing SRF or Elk-1 (HA tagged) separately or in combination (Fig 4). SRF or Elk1 were both able to induce the luciferase activity of the reporter (2 fold, *P* < 1.10^−3^) with a further increase when SRF and Elk-1 were combined with or without serum stimulation. To study the regulation of DUSP5 mRNA at the endogenous level, we performed siRNA silencing of SRF and Elk-1. SRF and Elk-1 siRNA transfection led to a significant decrease in DUSP5 mRNA induction levels after serum stimulation in comparison with the control situation, i.e. transfection with a control siRNA (Fig 5C). It is worth noting that SRF or Elk-1 siRNA did not completely abolish the DUSP5 mRNA serum induction. This could be explained by a lack of complete decrease of SRF or Elk-1 mRNA by the siRNA (Fig 5A and 5B). However, this induction was not anymore statistically significant.

**Fig 6:**
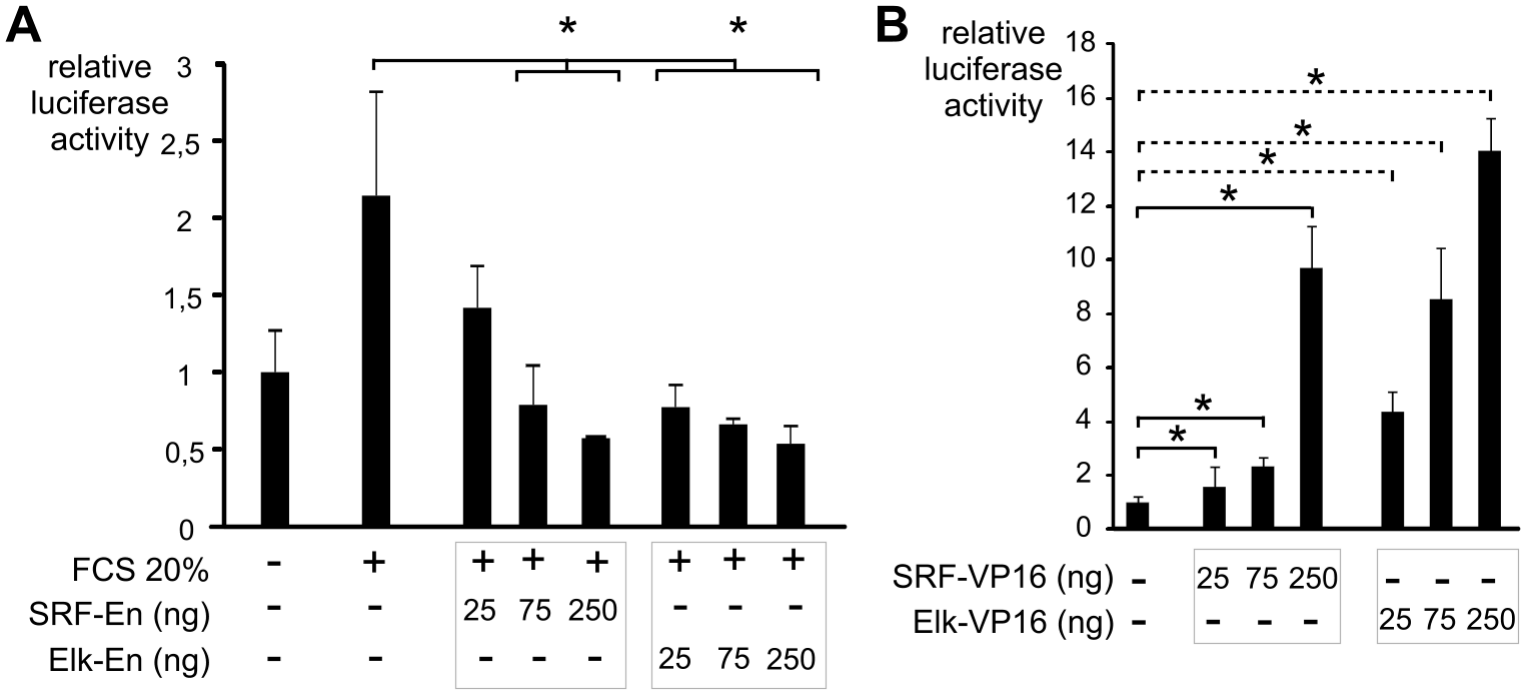
*DUSP5* promoter regulation by dominant negative and constitutively active expression vectors of SRF and Elk-1. NIH/3T3 cells were transiently transfected with 250 ng of *DUSP5* proximal promoter reporter in combination with the indicated expression vectors or an empty control vector. Luciferase assays were performed in triplicate and mean values ± S.D. are shown. (A) Additional transfected plasmids were the dominant negative SRF-En or Elk-En at increasing concentrations. Cells were starved (0.25% FCS) for 24 hours and then stimulated or not with 20% FCS for nine hours before assessment of luciferase activity. * *P* < 0.05. (B) Increasing concentrations of the constitutively active SRF-VP16 or Elk-VP16 was transfected. Cells were starved (0.25% FCS) for 24 hours before assessment of luciferase activity. * *P* < 0.05

**Fig 7:**
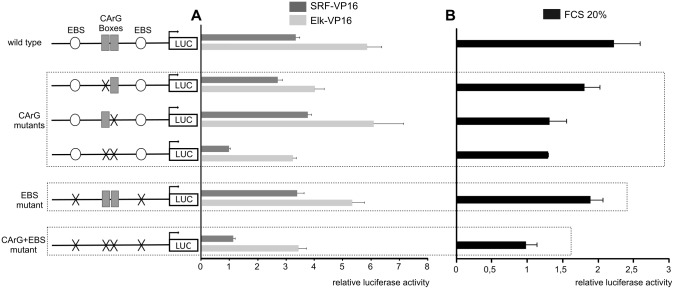
Role of CArG Boxes and EBS in *DUSP5* transcriptional regulation. Schematic representation of the wild type *DUSP5* proximal promoter is shown on the upper left part of the figure. Different reporter constructs with the indicated CArG Box and EBS mutated sites are illustrated below. Cells were transfected with 250 ng of each construct. At six hours post-transfection cells were serum-starved for 24 hours before assessment of luciferase activity. Luciferase assays were performed in triplicate and mean values ± S.D. are shown. (A) Cells were additionally transiently transfected with 200 ng of the constitutively active SRF-VP16 or Elk-VP16 or an empty control vector. (B) Alternatively cells were stimulated with 20% FCS for nine hours before assessment of luciferase activity, normalized to Renilla activity.

Experiments with plasmids expressing dominant negative forms of SRF (SRF-En) [28] or Elk-1 (Elk-En) [26] also support the implication of SRF and Elk-1. Serum-induced luciferase activity of the shortest *DUSP5* promoter reporter was decreased to control level by SRF-En and even more inhibited by Elk-En in a dose-dependent manner (Fig 6A). In Fig 6B the reverse experiment is reported: both dominant positive forms of SRF and Elk-1, SRF-VP16 [27] or Elk-VP16 [34], were able to induce the luciferase activity of the reporter, although with variable levels (see Figs 6B and 7A).

These results suggest that both SRF and Elk-1 may cooperate to induce the activity of *DUSP5* promoter.

### Mutational analyses of *DUSP5* promoter

To study in more detail promoter consensus sequences necessary and sufficient for regulation by serum, single or combined mutation of each CArG and EBS site were performed in the shortest *DUSP5* reporter vector and analyzed after transfection in NIH/3T3 cells. Mutations of the CArG boxes performed in our study were in agreement with a reported mutation [35, 36] resulting in the inability of binding endogenous SRF, and pointing to the crucial role of the central 6(A/T) for SRF DNA binding [37]. The putative two central core EBS (GGAA and GGAC respectively) were mutated to TTC as described previously [12, 38].

SRF-VP16 and Elk-VP16 induced luciferase activity of the wild type promoter, three- and six-fold, respectively (*P* < 0.05) (Fig 7A). Mutations of distal or proximal CArG each allowed the response to both dominant positive forms to occur. This result suggests that both CArG sites may be functional and able to bind SRF even though their activity does not seem to be additive. A combined mutation of the CArG sites reduced dramatically the response to both dominant positive constructs (*P* < 0.05): no induction by SRF-VP16 was observed and the induction by Elk-VP16 was reduced by 50%, indicating that, at least, one CArG site integrity is necessary for SRF action and required for optimal stimulation by Elk-VP16. Single mutation of each EBS did not influence the responses to SRF or Elk dominant positive forms (not shown). The same result was obtained with mutation of both EBS (Fig 7A), suggesting that Elk-1 may act even in the absence of binding to promoter sequences. When the four sites considered were disrupted, response to Elk-VP16 was significantly (*P* < 0.05) reduced to the level observed with the double mutant CArG (Fig 7A). The same panel of reporter vector was then tested for induction by the serum (Fig 7B). Mutation of distal CArG (CArG1) lightly inhibited (statistically insignificant) the serum response while the proximal (CArG2) and double CArG mutation (CARG1+2) almost abolished the serum effect (*P* < 0.05) (Fig 7B). This result suggests that the proximal CArG seems to play a predominant role as compared to the distal one. Single mutation of each EBS did not result in significant inhibitory effect (not shown). Mutation of both sites did not have any inhibitory effect either, suggesting again that Elk-1 DNA binding is not essential for transcription activation. Finally, mutation of the four sites completely counteracted the serum induction (*P* < 0.05) (Fig 7B).

**Fig 8:**
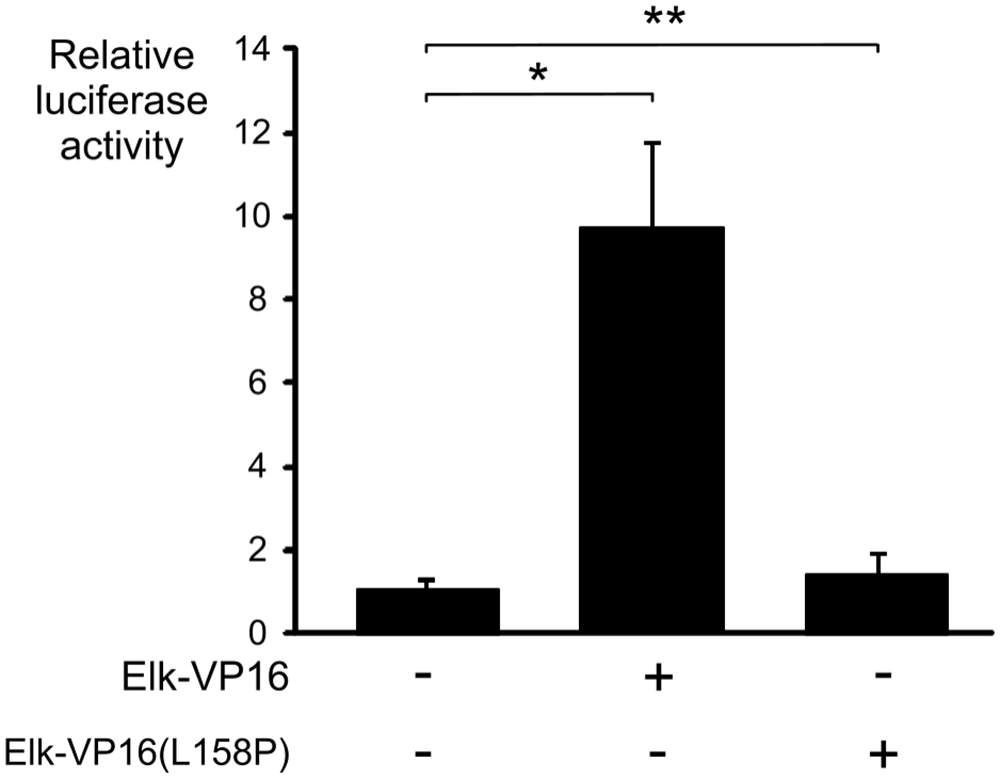
Elk-VP16(L158P) mutant is defective in *DUSP5* transcriptional activation. Serum starved (0.25%) NIH/3T3 cells were transiently transfected with 250 ng of *DUSP5* proximal promoter reporter and with 200 ng of the plasmids Elk-VP16 or Elk-VP16(L158P) before assessment of luciferase activity. Luciferase assays were performed in triplicate and mean values ± S.D. are shown. * *P* < 0.05, ** *P*: statistically insignificant.

Elk-1 can regulate the expression of target genes through SRF-independent and SRF-dependent mechanisms [39]. In the first case, Elk-1 can bind to high affinity with EBS independently from SRF and activate transcription. In the second case, specific interaction between the B-box region of Elk-1 and SRF is essential for transcriptional activation of Elk-1 target genes [30, 40] and it is known that leucine 158 is a crucial amino acid for such a contact [41]. The stimulation of *DUSP5* promoter activity was investigated in the presence of Elk-VP16 and Elk-VP16(L158P) mutant [26]: only the wild type Elk-VP16 construct able to interact with SRF produced an increase in luciferase activity demonstrating that an interaction between Elk-1 and SRF is required for *DUSP5* gene activation by Elk-VP16 (Fig 8).

### Binding of SRF and Elk-1 to *DUSP5* promoter sequences

**Fig 9:**
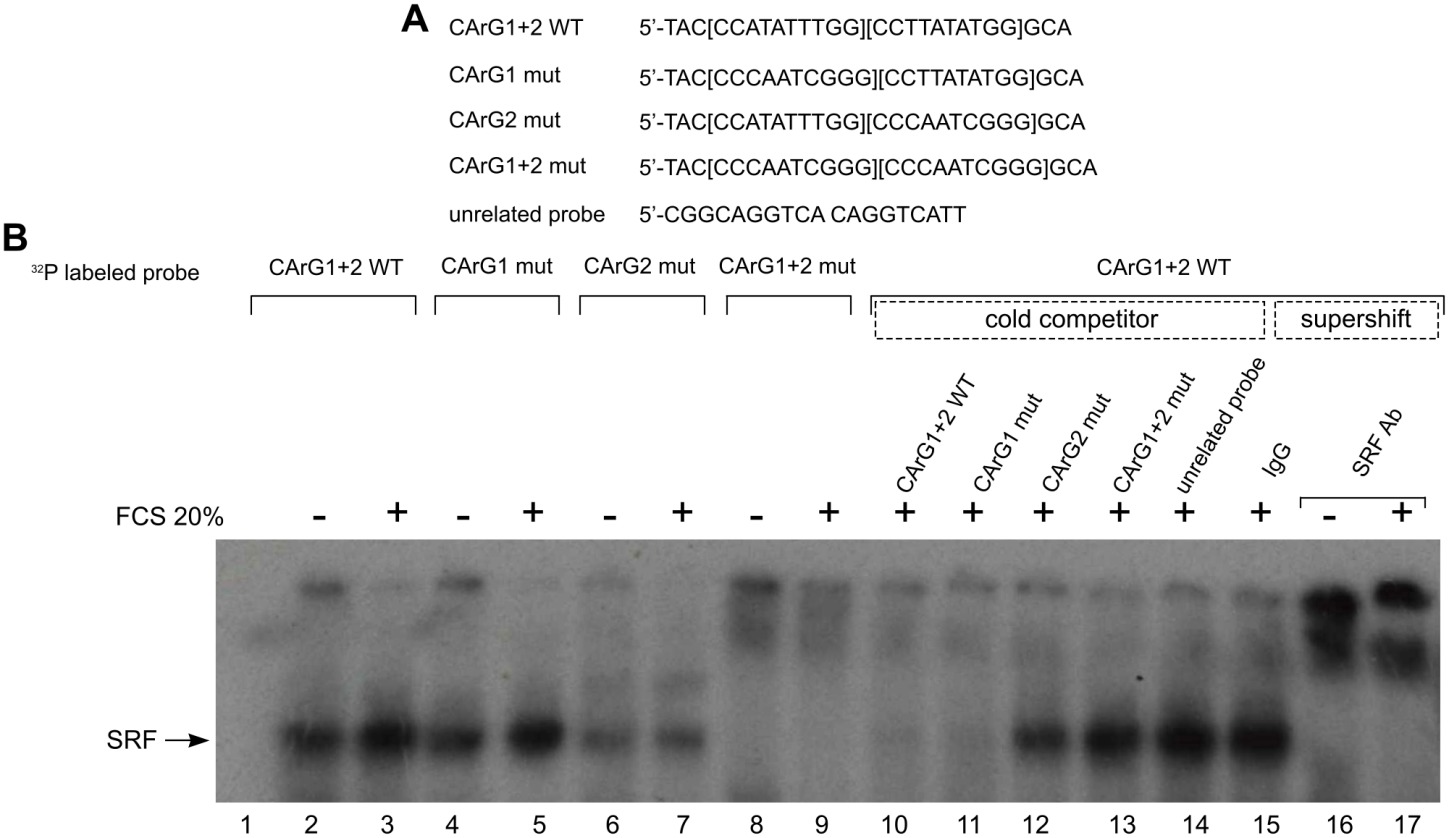
Proximal promoter region of *DUSP5* contains two functional CArG boxes. (A) Probes used in Electrophoretic Mobility Shift Assay (EMSA) containing CArG boxes of the proximal promoter region of *DUSP5* and an unrelated probe are shown. (B) EMSA was performed with the end labeled CArG boxes probe using 4 μg of nuclear extracts isolated from NIH/3T3 cells stimulated or not with 20% FCS for 30 minutes. For competition experiments, different unlabeled oligonucleotides (lane 10 to 14) were used. The indicated antibodies (lane 15 to 17) were used for supershift analysis.

EMSA experiments were performed to study the binding of SRF and Elk-1 to CArG boxes and putative EBS over the *DUSP5* promoter. 5’ ^32^P labelled oligonucleotides utilized to study the binding of SRF are represented in Fig 9A. Each probe was incubated with nuclear extracts from control or serum-treated cells, in presence or not of competitor oligonucleotide and of control or specific antibodies. The interaction was analyzed by non-denaturing gel electrophoresis and autoradiography (Fig 9B). A retarded band, suggesting binding of SRF, was detected in control and stimulated cell extracts with the wild type (WT) probe containing two CArG boxes (CArG1+2 WT, lane 2 and 3) and with the probe containing a mutation of the distal CArG (CArG1 mut) (lane 4 and 5). EMSA performed with serum-stimulated nuclear cell extracts resulted in a retarded band of increased intensity (compare lane 3 with lane 2 and lane 5 with lane 4) suggesting a higher DNA affinity and/or level of SRF following serum stimulation. The same band was of decreased intensity with the labelled probe mutated in the proximal CArG (CArG2 mut) (lane 6 and 7), indicating that the proximal SRE may have a better affinity for SRF, and plays a predominant role in serum response, according to our previous results (see Fig 7B). Competition with unlabelled excess of CArG1+2 WT (lane 10) and CArG1 mutated (lane 11) oligonucleotides was more efficient than with CArG2 mutated probe (lane 12). No competition was observed with a probe containing a mutation of both CArG boxes or an unrelated oligonucleotide (lane 13 and 14). Most importantly, in the retarded band, the presence of SRF in control and in stimulated cell extracts was demonstrated by the supershift with specific SRF antibody (lane 16 and 17), indicating that SRF may bind each CArG box of *DUSP5* promoter even in the absence of serum stimulation (lane 2, 4, and 6).

**Fig 10:**
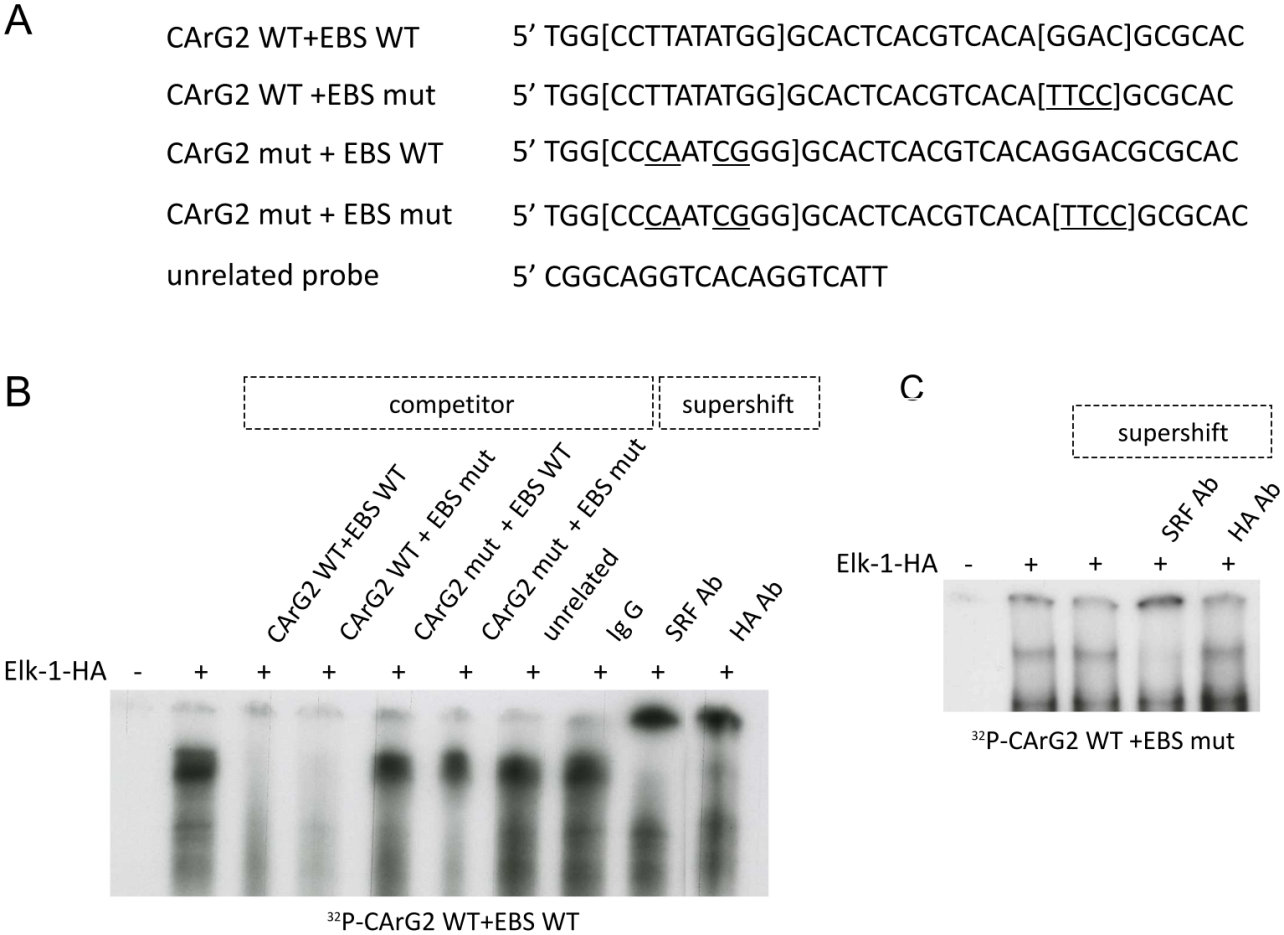
Proximal promoter region of *DUSP5* contains a functional serum responsive element. (A) DNA sequences of *DUSP5* promoter region containing proximal CArG box (CArG2) with proximal EBS and unrelated probe used in Electrophoretic Mobility Shift Assays (EMSA). (B) EMSA was performed with the end labelled probe containing wild type CArG Boxe and EBS using 5 μg of nuclear extracts isolated from NIH/3T3 transfected with hemagglutinin (HA) tagged Elk-1expression plasmid. For competition experiments, different unlabelled oligonucleotides (lane 3 to 7) were used. The indicated antibodies (lane 8 to 10) were used for supershift analysis. (C) EMSA was performed with the end labelled probe containing wild type CArG Boxe and mutated EBS using 5 μg of the same nuclear extracts. The indicated antibodies (lane 3 to 5) were used for supershift analysis.

Then the interaction of Elk-1 with promoter sequences was investigated. The probes that were used are listed in Fig 10A. They consisted of sequences including the CArG2 box (the most efficient for serum response) and the proximal putative EBS. Moreover, due to difficulties in obtaining a supershift with only endogenous Elk-1, the HA tagged Elk-1 expression plasmid [29] was transfected in NIH/3T3 cells used for preparation of nuclear extract. The EMSA experiment with a labelled probe containing WT CArG2 box and putative EBS (Fig 10B) showed a retarded band specifically competed by excess of WT oligonucleotide (lane 3), and by probe containing WT CArG box and mutant EBS (lane 3). On the contrary, the retarded band was not competed by CArG2 mutant (lane 5), the double mutant (lane 6), or the unrelated probe (lane 7). Moreover anti SRF (lane 9), anti HA (lane 10), antibodies produced a supershift indicating the presence of SRF and Elk-1 in a ternary complex with the WT oligonucleotide. The EMSA experiment with a labelled probe containing WT CArG2 box and mutated EBS (Fig 10C), showed a retarded band supershifted by anti SRF (lane 4), but not by anti HA antibody (lane 5). Altogether these results demonstrate the presence of SRF in the complex bound to the labelled probe, as expected with a WT CArG2 site. On the contrary Elk-1-HA is not found any more in the complex suggesting that the mutation created in the putative EBS site is deleterious for Elk-1 binding.

### SRF and Elk-1 bind to the endogenous *DUSP5* gene promoter

**Fig 11:**
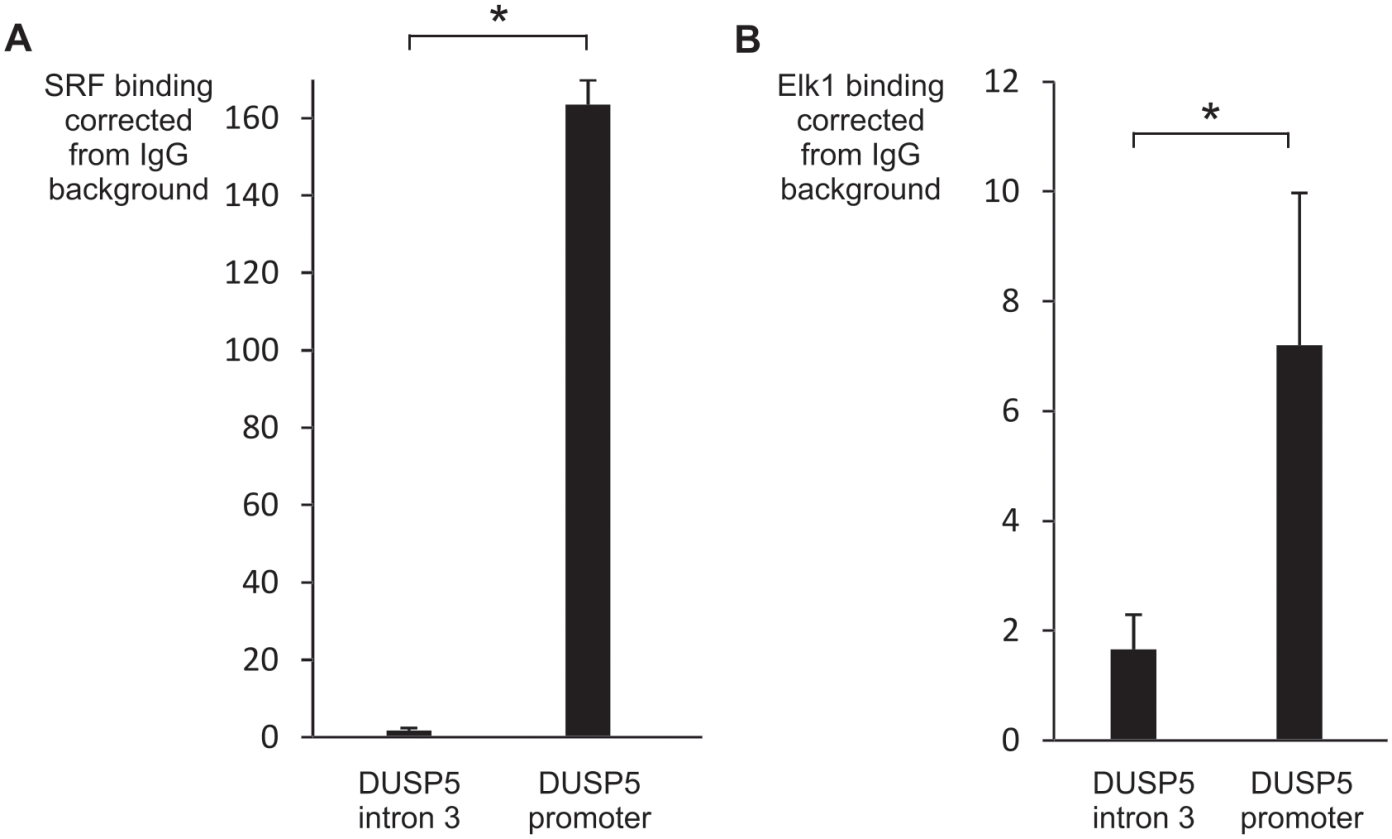
SRF and Elk-1 binding to the endogenous *DUSP5* promoter proximal region. ChIP assay was performed in NIH/3T3 cells stimulated for 30 minutes with 20% FCS after 24 hours of serum starvation, using an unrelated control antibody (IgG) and specific antibodies (A) for SRF or (B) Elk-1. Binding of SRF or Elk-1 to an intronic part of the *DUSP5* gene (negative control), and to the proximal part of the *DUSP5* promoter was measured by qPCR and corrected for background measured in IgG immunoprecipitates. Graphs show the mean and standard deviation of 3 independent experiments. * *P* < 0.05.

To further evaluate the binding of SRF and Elk-1 in the *DUSP5* proximal promoter region, we performed ChIP experiments (Fig 11). Using SRF (Fig 11A) and Elk-1 (Fig 11B) antibodies we demonstrated that SRF and Elk-1 were bound to the promoter region of *DUSP5* encompassing the two CArG boxes and the EBS. No significant enrichment was seen using primers amplifying the intron 3 of *DUSP5* gene (used as negative control).

## Discussion

In this study, we investigated the mechanisms involved in the regulation of *DUSP5* gene expression. First, we confirmed that *DUSP5* is an early response gene and that the MEK-ERK pathway is essentially involved in the regulation of *DUSP5* mRNA expression in NIH/3T3 cell line [10, 42, 43]. The Ras-ERK and PI3K-AKT pathways can negatively regulate each other’s activity [44]. AKT negatively regulates ERK activation. Such cross-inhibition is revealed when the PI3K pathway is chemically blocked by wortmannin, thereby releasing the cross-inhibition and effectively activating the MAPK pathway. In our work DUSP5 mRNA decreased in the presence of wortmannin after 60 minutes of FCS stimulation as observed by Tullai et al. [45] and then increased after 80 minutes. Tullai et al. reached the same conclusion of a predominant role for the MAPK pathway in the regulation of *DUSP5* expression and also found that PI3K pathway inhibition, as compared with induction with PDGF alone, decreased *DUSP5* mRNA levels of ~ 45% (30% in our work after 60 minutes of FCS stimulation) [45]. Nevertheless, DUSP5 protein levels do not seem to be affected by PI3K pathway inhibition [10].

We found that *DUSP5* gene expression is regulated at the transcriptional level and that *DUSP5* mRNA is not stabilized by the MEK-ERK pathway, contrary to *DUSP6* [25]. When MAPK pathway is inhibited *DUSP6* mRNA half-life is very short (about nine minutes) and ERK activation results in the stabilization of *DUSP6* mRNA (half-life ranging from 23 to 37 minutes) [25]. ERK is also able to bind to DUSP6 and cause its catalytic activation through the stabilization of the active phosphatase conformation [7–9]. On the contrary, in our work, *DUSP5* mRNA half-life remained unchanged after MAPK pathway inhibition. Previous work has shown that DUSP5 interacts with ERK and is responsible for its nuclear anchoring, but this binding is not accompanied by the catalytic activation of the phosphatase [2]. Furthermore, basal activity of DUSP5 is greater than that of DUSP6 before and even after its activation by ERK [2]. *DUSP5* mRNA half-life is also comparable to that of the early response gene c-fos mRNA (about 8 to 24 minutes according to the studied cell lines) [46]. The rapid induction and short half-life of *DUSP5* mRNA may lead to quick variation of DUSP5 protein level and enzymatic activity, responsible for a tight control of pERK levels.

We identified several EBS in the proximal and distal part of the *DUSP5* promoter sequence, which could provide a link with the MAPK pathway regulation through the binding of TCF. The presence of multiple binding motifs for ETS-domain transcription factors has previously been reported as a characteristic of Elk-1 target genes [33]. However, we showed that the proximal part of the *DUSP5* promoter is sufficient for its basal and serum or growth factor-induced activity, excluding a major role for EBS located in the distal part of the promoter.

We also identified in this proximal part two contiguous CArG boxes binding SRF. Tandem CArG elements have been described only for few genes, including SRF itself, whereas multiple non-contiguous CArG boxes in CArG containing genes are frequently found [47]. An analysis of the *DUSP5* promoter region using the Vertebrate Multiz Alignment & Conservation track [48] within the UCSC genome browser revealed that both core EBS sequences flanking the two contiguous CArG boxes are identical and almost in the same position in mouse, rat, and human. Two principal pathways regulate SRF differentially. The first one is RHO dependent, utilizes MRTF (myocardin-related transcription factors) family, and is regulated by the level of G-actin [49]. The second one is RAS dependent and, *via* ERK activation, SRF is involved in nucleoprotein complexes containing members of the TCF subfamily of the ETS domain transcriptional regulators, such as Elk-1 and recruited to EBS [49]. This pathway is illustrated by the paradigm of regulation of the early response gene c-fos where Elk-1 and SRF dimer form complexes on SRE which is composed of two binding sites (a CArG box for SRF and an EBS for Elk-1) and is involved in many cellular activities including cell growth and differentiation [50–54]. Binding of SRF to the CArG box with high affinity is required for the recruitment of one of the members of the TCF subfamily [52, 53, 55]. Among members of the ETS transcription factor family, Elk-1 seems to be a good candidate for the regulation of *DUSP5* gene expression for several reasons. Elk-1 is phosphorylated by ERK [36, 56, 57], thus providing an explanation for the induction of DUSP5 by the MAPK pathway signaling. ChIP-chip analysis highlighted that Elk-1-binding regions, mostly found within 1 kB of the transcription start site, are frequently co-bound by SRF, over the promoter of more than 200 genes. These co-occupied regions are more likely to be bound specifically by Elk-1 and not by other ETS-domain transcription factors, such as GABPA [33]. Furthermore, Nunes-Xavier et al. [18] tested the effect of RNA silencing of Ets-2 on *DUSP5* and *DUSP6* mRNA levels and found no inhibitory effect for *DUSP5* but only for *DUSP6*. In an extensive attempt to identify candidate transcription factor binding sites in genes regulated by specific signaling pathways in growth factor-stimulated human glioblastoma cell line, Tullai et al. [45] reported a predicted SRF binding sites in the *DUSP5* promoter. However, fully experimental validation of the putative CArG box remained to be provided.

As expected, the dominant negative SRF-En and Elk-En repressed *DUSP5* promoter activity. SRF-En has been shown to repress c-fos promoter activity and this effect was abolished by mutating the c-fos CArG box [28]. Elk-En has been shown to repress luciferase activity of an SRE reporter vector, containing both SRF binding site and an adjacent EBS motif [26]. Moreover, Elk-En is also able to repress the transcription of the SRF/TCF (Elk-1) c-fos-regulated gene but does not interfere with the expression of the Rho-actin SRF target gene *vinculin* [26]. On the other hand, dominant positive forms of SRF, SRF-VP16, and Elk-VP16 have been shown to efficiently activate a c-fos SRE-luc construct [34, 58]. In our work, the dominant positive Elk-VP16 and SRF-VP16 induced *DUSP5* promoter activity.

Our luciferase reporter assays with different mutants of the *DUSP5* promoter stimulated with serum suggest that the proximal CArG box may play a regulatory key role in *DUSP5* expression. Consistently, EMSA suggests that SRF may bind the proximal CArG box with higher affinity than the distal CArG. We suppose that only one of the two contiguous CArG boxes of the *DUSP5* promoter could play a predominant role *in vivo* because steric hindrance should exclude the possibility that both CArG boxes were bound by SRF homodimers [59, 60]. Moreover, in myotubes transduced with a constitutively active form of SRF (SRF-VP16), microarray data analyses revealed a statistically significant 1.4 fold increase in DUSP5 expression in comparison with the control situation, which is consistent with a role of SRF in the DUSP5 transcriptional regulation [61]. As expected, the mutation of both CArG boxes and EBS in the proximal promoter of *DUSP5* abolished the induction by the serum or SRF-VP16, but surprisingly not completely by Elk-VP16. Residual activations by Elk-VP16 of c-fos SRE reporter vectors containing ETS mutated binding site have already been reported in serum deprived NIH/3T3 cells [34, 35]. We have eliminated the possibility of creation of new responsive elements in the mutated promoters, which could bind Elk-1 or other transcription factors. The binding of Elk-1 to one or both EBS of the proximal *DUSP5* promoter gene does not seem to be essential to activate transcription, as suggested by induction of luciferase activity by the serum and Elk-VP16 in the context of double EBS mutation. On the contrary, the protein-protein interaction between SRF and Elk-1 seems to be essential for efficient ternary complex formation, as highlighted by the absence of induction of *DUSP5* promoter reporter with the mutant Elk-VP16 (L158P) which has lost its ability to bind SRF. Likewise, the mutant dominant negative Elk-En(L158P), which is not able to bind SRF either, has been shown to cause little repression of an SRE reporter vector (containing both SRF and EBS), but on the contrary can efficiently repress a reporter containing just ETS motifs [26]. Thus Elk-En(L158P) failed to repress SRE-mediated transcription but retained its ability to repress transcription from genes whose promoter regions are uniquely bound by Elk-1[26].

Our results suggest a model in which SRF is constitutively bound to the *DUSP5* promoter, as already proposed for c-fos [36]. Upon activation of the ERK pathway, Elk-1 is phosphorylated [56, 62] and forms a ternary complex with SRF over one SRE of the *DUSP5* promoter to activate transcription, without necessarily binding to the EBS. One previous ChIP-Seq study identified in a mammary human cell line the putative promoter region of *DUSP5* as significantly enriched in Elk-1 signal [63] in accordance with our data in favor of the role of Elk-1 in the transcriptional regulation of this gene.

We hypothesize that Elk-1 could interact directly with SRF, independently of its binding to EBS, as already described *in vitro* [64, 65]. A direct protein-protein interaction between the transcription factors Elk-1 and SRF, in the absence of the SRE, has been demonstrated previously, using pull-down assays [64]. Conventional ChIP technique using a single formaldehyde cross-linking step did not reproducibly demonstrate the presence of Elk-1 over the SRE of the *DUSP5* gene *in living cells* (data not shown). Using a ChIP method including a two-step cross-linking that first stabilizes large multiprotein complexes over DNA with DSG followed by the conventional formaldehyde cross-linking, we successfully demonstrated the presence of Elk-1 over the SRE of the *DUSP5* gene in NIH/3T3 cells. This pitfall supports the hypothesis of a direct protein-protein interaction between Elk-1 and SRF without necessarily DNA binding. Our EMSA performed with recombinant Elk-1-HA, demonstrated, *in vitro*, the presence of Elk-1 in the supershifted bands obtained with anti-HA antibody and suggested that the mutation created in the putative proximal EBS site (i.e. GGAC) of the DUSP5 promoter may be deleterious for Elk-1 binding. The putative core sequence of the proximal EBS site (i.e. GGAC) of the DUSP5 promoter identified by the TESS program has until recently only been described *in vitro* but not *in vivo* [21]. However others [66] identified a consensus Elk-1(5′-GGAC-3′) sequence within the neuregulin 3 (NRG3) promoter region using TFsearch software. Then they successfully precipitated this NRG 3 promoter region with anti-Elk-1 antibodies in ChIP assays of the nuclear fractions of HEK293 cells.

Furthermore our data suggesting that serum stimulation may result in increased binding capacity of SRF to CArG boxes is consistent with results previously reported [67–69]. Site specific phosphorylation of SRF by several kinases including pp90^rsk^ which is itself phosphorylated and activated by MAPK, has been shown to enhance the rate and affinity with which SRF associates with the SRE [67]. Increased DNA binding capacity does not seem to be explained by an increased dimerization of SRF, whereas change in the conformation of SRF that facilitates DNA binding could be an alternative explanation [69]. However, SRF mutants that cannot be phosphorylated were capable of activating transcription of SRF-dependent proliferation genes such as c-fos [65, 68]. Moreover, SRF level seems to remain unchanged after serum stimulation [70].

In summary, we have shown that the DUSP5 phosphatase is regulated at the transcriptional level by the MAPK pathway and that SRE is involved in the regulation of this early response gene. We propose a model in which SRF is bound to *DUSP5* promoter in the basal state, and Elk-1 could also be recruited at the *DUSP5* promoter through direct association with SRF regardless of its DNA-binding or through its binding site and activate transcription. Thus induction of DUSP5 by the MEK-ERK pathway serves as an important feedback loop that controls activation of ERK1/2.

## References

[1] Chang L, Karin M. Mammalian MAP kinase signalling cascades. Nature. 2001;410(6824):37–40. . 11242034 10.1038/35065000

[2] Mandl M, Slack DN, Keyse SM. Specific inactivation and nuclear anchoring of extracellular signal-regulated kinase 2 by the inducible dual-specificity protein phosphatase DUSP5. Mol Cell Biol. 2005;25(5):1830–45. . 15713638 10.1128/MCB.25.5.1830-1845.2005 PMC549372

[3] Groom LA, Sneddon AA, Alessi DR, Dowd S, Keyse SM. Differential regulation of the MAP, SAP and RK/p38 kinases by Pyst1, a novel cytosolic dual-specificity phosphatase. Embo J. 1996;15(14):3621–32. . 8670865 PMC451978

[4] Muda M, Theodosiou A, Gillieron C, Smith A, Chabert C, Camps M, et al The mitogen-activated protein kinase phosphatase-3 N-terminal noncatalytic region is responsible for tight substrate binding and enzymatic specificity. J Biol Chem. 1998;273(15):9323–9. . 9535927 10.1074/jbc.273.15.9323

[5] Arkell RS, Dickinson RJ, Squires M, Hayat S, Keyse SM, Cook SJ. DUSP6/MKP-3 inactivates ERK1/2 but fails to bind and inactivate ERK5. Cell Signal. 2008;20(5):836–43. 10.1016/j.cellsig.2007.12.014 18280112

[6] Keyse SM. Protein phosphatases and the regulation of mitogen-activated protein kinase signalling. Curr Opin Cell Biol. 2000;12(2):186–92. . 10712927 10.1016/s0955-0674(99)00075-7

[7] Karlsson M, Mathers J, Dickinson RJ, Mandl M, Keyse SM. Both nuclear-cytoplasmic shuttling of the dual specificity phosphatase MKP-3 and its ability to anchor MAP kinase in the cytoplasm are mediated by a conserved nuclear export signal. J Biol Chem. 2004;279(40):41882–91. . 15269220 10.1074/jbc.M406720200

[8] Fjeld CC, Rice AE, Kim Y, Gee KR, Denu JM. Mechanistic basis for catalytic activation of mitogen-activated protein kinase phosphatase 3 by extracellular signal-regulated kinase. J Biol Chem. 2000;275(10):6749–57. . 10702230 10.1074/jbc.275.10.6749

[9] Dowd S, Sneddon AA, Keyse SM. Isolation of the human genes encoding the pyst1 and Pyst2 phosphatases: characterisation of Pyst2 as a cytosolic dual-specificity MAP kinase phosphatase and its catalytic activation by both MAP and SAP kinases. Journal of cell science. 1998;111 (Pt 22):3389–99. . 9788880 10.1242/jcs.111.22.3389

[10] Kucharska A, Rushworth LK, Staples C, Morrice NA, Keyse SM. Regulation of the inducible nuclear dual-specificity phosphatase DUSP5 by ERK MAPK. Cell Signal. 2009;21(12):1794–805. 10.1016/j.cellsig.2009.07.015 19666109

[11] Zhang Z, Kobayashi S, Borczuk AC, Leidner RS, Laframboise T, Levine AD, et al Dual specificity phosphatase 6 (DUSP6) is an ETS-regulated negative feedback mediator of oncogenic ERK signaling in lung cancer cells. Carcinogenesis. 2010;31(4):577–86. 10.1093/carcin/bgq020 20097731 PMC2847094

[12] Ekerot M, Stavridis MP, Delavaine L, Mitchell MP, Staples C, Owens DM, et al Negative-feedback regulation of FGF signalling by DUSP6/MKP-3 is driven by ERK1/2 and mediated by Ets factor binding to a conserved site within the DUSP6/MKP-3 gene promoter. Biochem J. 2008;412(2):287–98. 10.1042/BJ20071512 18321244 PMC2474557

[13] Bermudez O, Pages G, Gimond C. The dual-specificity MAP kinase phosphatases: critical roles in development and cancer. American journal of physiology Cell physiology. 2010;299(2):C189–202. 10.1152/ajpcell.00347.2009 . 20463170

[14] Knauf JA, Fagin JA. Role of MAPK pathway oncoproteins in thyroid cancer pathogenesis and as drug targets. Curr Opin Cell Biol. 2009;21(2):296–303. 10.1016/j.ceb.2009.01.013 . 19231149

[15] Griffith OL, Melck A, Jones SJ, Wiseman SM. Meta-analysis and meta-review of thyroid cancer gene expression profiling studies identifies important diagnostic biomarkers. Journal of clinical oncology: official journal of the American Society of Clinical Oncology. 2006;24(31):5043–51. 10.1200/JCO.2006.06.7330 . 17075124

[16] Lee JU, Huang S, Lee MH, Lee SE, Ryu MJ, Kim SJ, et al Dual specificity phosphatase 6 as a predictor of invasiveness in papillary thyroid cancer. European journal of endocrinology / European Federation of Endocrine Societies. 2012;167(1):93–101. 10.1530/EJE-12-0010 . 22535643

[17] Cancer Genome Atlas Research N. Integrated genomic characterization of papillary thyroid carcinoma. Cell. 2014;159(3):676–90. 10.1016/j.cell.2014.09.050 25417114 PMC4243044

[18] Nunes-Xavier CE, Tarrega C, Cejudo-Marin R, Frijhoff J, Sandin A, Ostman A, et al Differential up-regulation of MAP kinase phosphatases MKP3/DUSP6 and DUSP5 by Ets2 and c-Jun converge in the control of the growth arrest versus proliferation response of MCF-7 breast cancer cells to phorbol ester. J Biol Chem. 2010;285(34):26417–30. 10.1074/jbc.M110.121830 20554528 PMC2924073

[19] Ueda K, Arakawa H, Nakamura Y. Dual-specificity phosphatase 5 (DUSP5) as a direct transcriptional target of tumor suppressor p53. Oncogene. 2003;22(36):5586–91. 10.1038/sj.onc.1206845 . 12944906

[20] Furukawa T, Tanji E, Xu S, Horii A. Feedback regulation of DUSP6 transcription responding to MAPK1 via ETS2 in human cells. Biochem Biophys Res Commun. 2008;377(1):317–20. 10.1016/j.bbrc.2008.10.003 18848526

[21] Hollenhorst PC, McIntosh LP, Graves BJ. Genomic and biochemical insights into the specificity of ETS transcription factors. Annu Rev Biochem. 2011;80:437–71. 10.1146/annurev.biochem.79.081507.103945 21548782 PMC5568663

[22] Foulds CE, Nelson ML, Blaszczak AG, Graves BJ. Ras/mitogen-activated protein kinase signaling activates Ets-1 and Ets-2 by CBP/p300 recruitment. Mol Cell Biol. 2004;24(24):10954–64. 10.1128/MCB.24.24.10954-10964.2004 15572696 PMC533975

[23] McCarthy SA, Chen D, Yang BS, Garcia Ramirez JJ, Cherwinski H, Chen XR, et al Rapid phosphorylation of Ets-2 accompanies mitogen-activated protein kinase activation and the induction of heparin-binding epidermal growth factor gene expression by oncogenic Raf-1. Mol Cell Biol. 1997;17(5):2401–12. 9111309 10.1128/mcb.17.5.2401 PMC232089

[24] Yang BS, Hauser CA, Henkel G, Colman MS, Van Beveren C, Stacey KJ, et al Ras-mediated phosphorylation of a conserved threonine residue enhances the transactivation activities of c-Ets1 and c-Ets2. Mol Cell Biol. 1996;16(2):538–47. 8552081 10.1128/mcb.16.2.538 PMC231032

[25] Bermudez O, Jouandin P, Rottier J, Bourcier C, Pages G, Gimond C. Post-transcriptional regulation of the DUSP6/MKP-3 phosphatase by MEK/ERK signaling and hypoxia. J Cell Physiol. 2011;226(1):276–84. 10.1002/jcp.22339 20665674

[26] Vickers ER, Kasza A, Kurnaz IA, Seifert A, Zeef LA, O'Donnell A, et al Ternary complex factor-serum response factor complex-regulated gene activity is required for cellular proliferation and inhibition of apoptotic cell death. Mol Cell Biol. 2004;24(23):10340–51. . 15542842 10.1128/MCB.24.23.10340-10351.2004 PMC529045

[27] Dalton S, Treisman R. Characterization of SAP-1, a protein recruited by serum response factor to the c-fos serum response element. Cell. 1992;68(3):597–612. . 1339307 10.1016/0092-8674(92)90194-h

[28] Stritt C, Stern S, Harting K, Manke T, Sinske D, Schwarz H, et al Paracrine control of oligodendrocyte differentiation by SRF-directed neuronal gene expression. Nat Neurosci. 2009;12(4):418–27. 10.1038/nn.2280 19270689

[29] Vanhoutte P, Nissen JL, Brugg B, Gaspera BD, Besson MJ, Hipskind RA, et al Opposing roles of Elk-1 and its brain-specific isoform, short Elk-1, in nerve growth factor-induced PC12 differentiation. J Biol Chem. 2001;276(7):5189–96. . 11050086 10.1074/jbc.M006678200

[30] Treisman R, Marais R, Wynne J. Spatial flexibility in ternary complexes between SRF and its accessory proteins. EMBO J. 1992;11(12):4631–40. 1425594 10.1002/j.1460-2075.1992.tb05565.x PMC557039

[31] Zeliadt NA, Mauro LJ, Wattenberg EV. Reciprocal regulation of extracellular signal regulated kinase 1/2 and mitogen activated protein kinase phosphatase-3. Toxicology and applied pharmacology. 2008;232(3):408–17. 10.1016/j.taap.2008.08.007 18771677 PMC2581931

[32] Bucher P. Weight matrix descriptions of four eukaryotic RNA polymerase II promoter elements derived from 502 unrelated promoter sequences. Journal of molecular biology. 1990;212(4):563–78. 10.1016/0022-2836(90)90223-9 . 2329577

[33] Boros J, Donaldson IJ, O'Donnell A, Odrowaz ZA, Zeef L, Lupien M, et al Elucidation of the ELK1 target gene network reveals a role in the coordinate regulation of core components of the gene regulation machinery. Genome Res. 2009;19(11):1963–73. 10.1101/gr.093047.109 19687146 PMC2775591

[34] Price MA, Rogers AE, Treisman R. Comparative analysis of the ternary complex factors Elk-1, SAP-1a and SAP-2 (ERP/NET). Embo J. 1995;14(11):2589–601. . 7540136 10.1002/j.1460-2075.1995.tb07257.x PMC398373

[35] Hill CS, Wynne J, Treisman R. Serum-regulated transcription by serum response factor (SRF): a novel role for the DNA binding domain. Embo J. 1994;13(22):5421–32. . 7957108 10.1002/j.1460-2075.1994.tb06877.x PMC395499

[36] Hill CS, Marais R, John S, Wynne J, Dalton S, Treisman R. Functional analysis of a growth factor-responsive transcription factor complex. Cell. 1993;73(2):395–406. . 8477450 10.1016/0092-8674(93)90238-l

[37] Pellegrini L, Tan S, Richmond TJ. Structure of serum response factor core bound to DNA. Nature. 1995;376(6540):490–8. . 7637780 10.1038/376490a0

[38] Withers DA, Hakomori SI. Human alpha (1,3)-fucosyltransferase IV (FUTIV) gene expression is regulated by elk-1 in the U937 cell line. J Biol Chem. 2000;275(51):40588–93. . 11006292 10.1074/jbc.M007262200

[39] Shaw PE, Saxton J. Ternary complex factors: prime nuclear targets for mitogen-activated protein kinases. The international journal of biochemistry & cell biology. 2003;35(8):1210–26. . 12757758 10.1016/s1357-2725(03)00031-1

[40] Janknecht R, Nordheim A. Elk-1 protein domains required for direct and SRF-assisted DNA-binding. Nucleic acids research. 1992;20(13):3317–24. 1630903 10.1093/nar/20.13.3317 PMC312483

[41] Ling Y, Lakey JH, Roberts CE, Sharrocks AD. Molecular characterization of the B-box protein-protein interaction motif of the ETS-domain transcription factor Elk-1. EMBO J. 1997;16(9):2431–40. 10.1093/emboj/16.9.2431 9171356 PMC1169843

[42] Ishibashi T, Bottaro DP, Michieli P, Kelley CA, Aaronson SA. A novel dual specificity phosphatase induced by serum stimulation and heat shock. J Biol Chem. 1994;269(47):29897–902. . 7961985

[43] Kwak SP, Dixon JE. Multiple dual specificity protein tyrosine phosphatases are expressed and regulated differentially in liver cell lines. J Biol Chem. 1995;270(3):1156–60. . 7836374 10.1074/jbc.270.3.1156

[44] Mendoza MC, Er EE, Blenis J. The Ras-ERK and PI3K-mTOR pathways: cross-talk and compensation. Trends in biochemical sciences. 2011;36(6):320–8. 10.1016/j.tibs.2011.03.006 21531565 PMC3112285

[45] Tullai JW, Schaffer ME, Mullenbrock S, Kasif S, Cooper GM. Identification of transcription factor binding sites upstream of human genes regulated by the phosphatidylinositol 3-kinase and MEK/ERK signaling pathways. J Biol Chem. 2004;279(19):20167–77. . 14769801 10.1074/jbc.M309260200

[46] Kabnick KS, Housman DE. Determinants that contribute to cytoplasmic stability of human c-fos and beta-globin mRNAs are located at several sites in each mRNA. Mol Cell Biol. 1988;8(8):3244–50. 3211141 10.1128/mcb.8.8.3244-3250.1988 PMC363556

[47] Sun Q, Chen G, Streb JW, Long X, Yang Y, Stoeckert CJ Jr., et al Defining the mammalian CArGome. Genome Res. 2006;16(2):197–207. 10.1101/gr.4108706 16365378 PMC1361715

[48] Blanchette M, Kent WJ, Riemer C, Elnitski L, Smit AF, Roskin KM, et al Aligning multiple genomic sequences with the threaded blockset aligner. Genome Res. 2004;14(4):708–15. 10.1101/gr.1933104 15060014 PMC383317

[49] Posern G, Treisman R. Actin' together: serum response factor, its cofactors and the link to signal transduction. Trends Cell Biol. 2006;16(11):588–96. . 17035020 10.1016/j.tcb.2006.09.008

[50] Chai J, Tarnawski AS. Serum response factor: discovery, biochemistry, biological roles and implications for tissue injury healing. J Physiol Pharmacol. 2002;53(2):147–57. . 12120892

[51] Konig H, Ponta H, Rahmsdorf U, Buscher M, Schonthal A, Rahmsdorf HJ, et al Autoregulation of fos: the dyad symmetry element as the major target of repression. Embo J. 1989;8(9):2559–66. . 2511006 10.1002/j.1460-2075.1989.tb08394.x PMC401256

[52] Shaw PE, Schroter H, Nordheim A. The ability of a ternary complex to form over the serum response element correlates with serum inducibility of the human c-fos promoter. Cell. 1989;56(4):563–72. . 2492906 10.1016/0092-8674(89)90579-5

[53] Hipskind RA, Rao VN, Mueller CG, Reddy ES, Nordheim A. Ets-related protein Elk-1 is homologous to the c-fos regulatory factor p62TCF. Nature. 1991;354(6354):531–4. . 1722028 10.1038/354531a0

[54] Sharrocks AD. Complexities in ETS-domain transcription factor function and regulation: lessons from the TCF (ternary complex factor) subfamily. The Colworth Medal Lecture. Biochem Soc Trans. 2002;30(2):1–9. . 12023815 10.1042/

[55] Hassler M, Richmond TJ. The B-box dominates SAP-1-SRF interactions in the structure of the ternary complex. EMBO J. 2001;20(12):3018–28. 10.1093/emboj/20.12.3018 11406578 PMC150215

[56] Marais R, Wynne J, Treisman R. The SRF accessory protein Elk-1 contains a growth factor-regulated transcriptional activation domain. Cell. 1993;73(2):381–93. . 8386592 10.1016/0092-8674(93)90237-k

[57] Janknecht R, Ernst WH, Pingoud V, Nordheim A. Activation of ternary complex factor Elk-1 by MAP kinases. EMBO J. 1993;12(13):5097–104. 8262053 10.1002/j.1460-2075.1993.tb06204.x PMC413771

[58] Schratt G, Philippar U, Hockemeyer D, Schwarz H, Alberti S, Nordheim A. SRF regulates Bcl-2 expression and promotes cell survival during murine embryonic development. EMBO J. 2004;23(8):1834–44. 10.1038/sj.emboj.7600188 15057274 PMC394242

[59] Norman C, Runswick M, Pollock R, Treisman R. Isolation and properties of cDNA clones encoding SRF, a transcription factor that binds to the c-fos serum response element. Cell. 1988;55(6):989–1003. . 3203386 10.1016/0092-8674(88)90244-9

[60] Mo Y, Ho W, Johnston K, Marmorstein R. Crystal structure of a ternary SAP-1/SRF/c-fos SRE DNA complex. Journal of molecular biology. 2001;314(3):495–506. 10.1006/jmbi.2001.5138 . 11846562

[61] Guerci A, Lahoute C, Hebrard S, Collard L, Graindorge D, Favier M, et al Srf-dependent paracrine signals produced by myofibers control satellite cell-mediated skeletal muscle hypertrophy. Cell metabolism. 2012;15(1):25–37. 10.1016/j.cmet.2011.12.001 . 22225874

[62] Gille H, Sharrocks AD, Shaw PE. Phosphorylation of transcription factor p62TCF by MAP kinase stimulates ternary complex formation at c-fos promoter. Nature. 1992;358(6385):414–7. 10.1038/358414a0 . 1322499

[63] Odrowaz Z, Sharrocks AD. ELK1 uses different DNA binding modes to regulate functionally distinct classes of target genes. PLoS genetics. 2012;8(5):e1002694 10.1371/journal.pgen.1002694 22589737 PMC3349735

[64] Shore P, Sharrocks AD. The transcription factors Elk-1 and serum response factor interact by direct protein-protein contacts mediated by a short region of Elk-1. Mol Cell Biol. 1994;14(5):3283–91. 8164681 10.1128/mcb.14.5.3283-3291.1994 PMC358695

[65] Iyer D, Chang D, Marx J, Wei L, Olson EN, Parmacek MS, et al Serum response factor MADS box serine-162 phosphorylation switches proliferation and myogenic gene programs. Proceedings of the National Academy of Sciences of the United States of America. 2006;103(12):4516–21. 10.1073/pnas.0505338103 16537394 PMC1450203

[66] Plani-Lam JH, Chow TC, Siu KL, Chau WH, Ng MH, Bao S, et al PTPN21 exerts pro-neuronal survival and neuritic elongation via ErbB4/NRG3 signaling. The international journal of biochemistry & cell biology. 2015;61:53–62. 10.1016/j.biocel.2015.02.003 . 25681686

[67] Rivera VM, Miranti CK, Misra RP, Ginty DD, Chen RH, Blenis J, et al A growth factor-induced kinase phosphorylates the serum response factor at a site that regulates its DNA-binding activity. Mol Cell Biol. 1993;13(10):6260–73. 8413226 10.1128/mcb.13.10.6260-6273.1993 PMC364685

[68] Janknecht R, Hipskind RA, Houthaeve T, Nordheim A, Stunnenberg HG. Identification of multiple SRF N-terminal phosphorylation sites affecting DNA binding properties. EMBO J. 1992;11(3):1045–54. 1547771 10.1002/j.1460-2075.1992.tb05143.x PMC556545

[69] Manak JR, Prywes R. Mutation of serum response factor phosphorylation sites and the mechanism by which its DNA-binding activity is increased by casein kinase II. Mol Cell Biol. 1991;11(7):3652–9. 2046671 10.1128/mcb.11.7.3652-3659.1991 PMC361119

[70] Manak JR, Prywes R. Phosphorylation of serum response factor by casein kinase II: evidence against a role in growth factor regulation of fos expression. Oncogene. 1993;8(3):703–11. . 8437853

